# Size diversity in Swiss Bronze Age cattle

**DOI:** 10.1002/oa.2654

**Published:** 2018-04-17

**Authors:** M. Bopp‐Ito, S. Deschler‐Erb, W. Vach, J. Schibler

**Affiliations:** ^1^ Integrative Prehistory and Archaeological Science University of Basel Basel Switzerland; ^2^ Institute of Medical Biometry and Statistics, Medical Faculty and Medical Center, University of Freiburg Freiburg Germany; ^3^ Department of Orthopaedics and Traumatology University Hospital Basel Basel Switzerland

**Keywords:** bronze age, cattle husbandry, logarithmic size index, osteometry, sex ratio, zooarchaeology

## Abstract

To date, osteometric data for Swiss Bronze Age cattle, particularly from Alpine sites, are scarce. In the present study, using a large dataset generated by combining preexisting data with recent data obtained from a large Alpine site, cattle size from the Late Neolithic to the Late Bronze Age (LBA) in populations from different sites and regions was evaluated using the logarithmic size index and other statistical analysis. Additionally, the finite mixture model and a meta‐analytic technique were used to observe possible effects of sex ratios on cattle size. Results indicated that sex ratios did not affect size distribution. Cattle populations did not differ over time, but the Alpine cattle were smaller than the Central Plateau cattle. There were two distinct sizes in the Alpine cattle populations. It is suggested that the different economic interrelationships between Alpine and other geographically related communities might have led to the emergence of size diversity in Swiss Bronze Age cattle. Further interdisciplinary studies with larger sample sizes are required to confirm these possibilities.

## 1 INTRODUCTION

Specimens identified in archaeological sites suggest that cattle were the main domestic animal (approximately 30%–70% in each site) during the Bronze Age (2200–800 BC) in Switzerland, and it is presumed that they were exploited for meat and milk and as working power (Bopp‐Ito, 2012; Plüss, 2011; Schibler, 2017; Schibler & Studer, 1998; Stopp, 2015). Cattle size in the Swiss Bronze Age was smaller than that in the Neolithic and Roman periods (Hüster Plogmann & Schibler, 1997; Schibler & Schlumbaum, 2007). However, little is known about the change in cattle size during the Bronze Age, and the diversity in cattle size distribution across regions, especially across the Central Plateau (hereafter calls Plateau) and the Alpine regions, has not been explored owing to the scarcity of osteometric data from Alpine sites.

The Alpine sites, which underwent colonization related to lithic raw materials during the Late Neolithic (LN) or Copper Age (Della Casa, 2003) and had expanded settlements with unique culture during the Early Bronze Age (EBA), and Plateau sites, which were sequenced since the Early Neolithic with agropastoral subsistence (Schibler, 2017), were located at different economic, geographical, topographical, and environmental crossroads (Jecker, 2015; Jennings, 2015; Reitmaier, 2012; Rychner, 1998a; Stöckli, 1995). Animal husbandry was influenced by these factors: for example, while Plateau settlements exploited cattle for secondary products from the second half of the fourth millennium BC onwards (Deschler‐Erb & Marti‐Grädel, 2004; Hüster Plogmann & Schibler, 1997), exploitation for secondary products at the Alpine settlements might have been intensified during the LBA (Bopp‐Ito, 2012; Plüss, 2011; Stopp, 2015).

Previous studies have proposed plausible factors that could have had an impact on animal morphologies and caused size variations, such as livestock diet (Breuer, Rehazek, & Stopp, 1999), which was linked to climate (Davis, 1981) or altitude (Knockaert et al., 2017), breeding strategies (Duval, Horard‐Herbin, & Lepetz, 2013; Trixl, Steidl, & Peters, 2017), idiosyncratic choices of husbandry (Cucchi et al., 2016), introduction of new animal forms (Gaastra, 2014; MacKinnon, 2010), transalpine mobility, and migration of humans together with their livestock (Grupe, Hölzl, Mayr, & Söllner, 2017), or selection of specific sex, such as small female cattle (Manning, Timpson, Shennan, & Crema, 2015). Because body size and sex are strongly correlated (Davis et al., 2012) and sex ratio provides a hint of cattle exploitation, computation of sex ratios of cattle populations was considered necessary.

Recently, substantial Bronze Age osteometric data were acquired from an Alpine settlement at Savognin‐Padnal (hereafter calls Padnal) (Bopp‐Ito, 2012), which might have been near the copper mining site for bronze production (Della Casa, Naef, & Turck, 2016; Rageth, 1986). Thus, the aim of this study was to shed new light on the change and diversity of cattle size linked with husbandry practices across settlements during the Bronze Age. The analyses of cattle size and sex ratios as preconditions may provide evidence on possible influences of sex ratio on size diversity. Samples from the aforementioned different regional sites would address factors that might have had the most influence on cattle size diversity.

## 2 MATERIALS AND METHODS

### 2.1 Grouping of the studied samples by assemblages, regions, and periods

**Figure 1:**
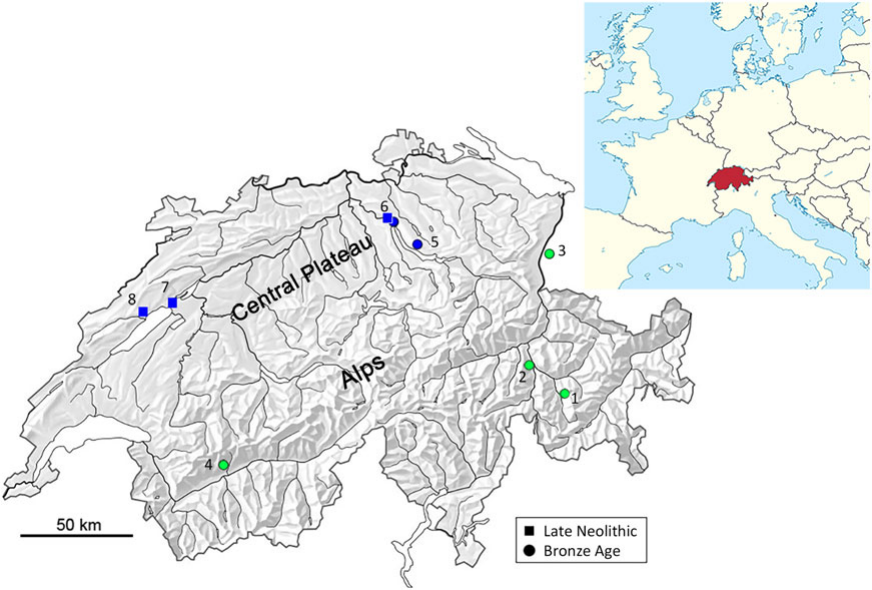
Locations of the studied sites between Late Neolithic (square) and Bronze Age (dot) periods in Switzerland and Liechtenstein. Light gray (Green) dots (Nos. 1, 2, 3, and 4) correspond to the Alpine Bronze Age sites, dark gray (blue) dots (Nos. 5 and 6) to the Central Plateau Bronze Age sites, and dark gray (blue) squares (Nos. 6, 7, and 8) to the Central Plateau Late Neolithic sites. Numbers refer to the entries in Table 1: 1 = Savognin‐Padnal; 2 = Cresta‐Cazis; 3 = Schellenberg‐Borscht; 4 = Ayent Le Château; 5 = Meilen‐Obermeilen; 6 = Zürich sites; 7 = Pont‐de‐Thielle; 8 = Auvernier sites [Colour figure can be viewed at http://wileyonlinelibrary.com]

Combining the data on osteometric samples obtained from four assemblages (Horizont [synonymous with layer] B, C, D, E) in Padnal (Bopp‐Ito, unpublished data) with preexisting data of samples obtained from 32 assemblages from LN (ca. 2800–2200 bc), EBA (2200–1600 bc), Middle Bronze Age (MBA; 1600–1300 bc), and LBA (1300–800 BC; Rychner, 1998b), sites of modern‐day Switzerland and Liechtenstein, created a large sample size (total assemblages *n* = 36; Figure 1, Table 1, Table S1, and Appendices S1 and S2). Although cattle specimens were decreased at the Plateau sites, the ones at the Alpine sites were increased (Schibler, 2017; Figure S1); in particular, the senile adult cattle at the Alpine sites increased during the LBA (Bopp‐Ito, 2012; Plüss, 2011; Stopp, 2015). In contrast, dominant senile Plateau cattle appeared between the LN and EBA (Hüster Plogmann & Schibler, 1997; Figure S2).

**Table 1:** Sample sources for the Late Neolithic and Bronze Age cattle excavated from four Alpine sites (18 assemblages) and nine Central Plateau sites (18 assemblages) in Switzerland and Liechtenstein. The map number (No.) corresponds to the locations shown in Figure 1. For details, see Appendix S1

Region	Period	No.	Assemblage	Reference	Year	LSI *n*	Mc Bd *n*
Alpine	Late Bronze Age	4	Ayent Le Château	Chaix	1990	1	
		2	Cresta‐Cazis, Planum 14	Plüss	2007	10	1
		1	Savognin‐Padnal, Horizont B	Bopp‐Ito	Unpub.	39	10
	Total					50	11
	Middle Bronze Age	2	Cresta‐Cazis, Planum 12	Plüss	2007	22	2
		2	Cresta‐Cazis, Planum 11	Plüss	2007	5	1
		2	Cresta‐Cazis, Planum 10	Plüss	2007	3	1
		1	Savognin‐Padnal, Horizont C	Bopp‐Ito	Unpub.	12	2
		1	Savognin‐Padnal, Horizont D	Bopp‐Ito	Unpub.	13	1
	Total					55	7
	Early Bronze Age	4	Ayent Le Château	Chaix	1990	1	
		2	Cresta‐Cazis, Planum 8	Plüss	2007	15	
		2	Cresta‐Cazis, Planum 5	Plüss	2007	4	
		2	Cresta‐Cazis, Planum 4	Plüss	2007	3	
		2	Cresta‐Cazis, Planum 3	Plüss	2007	13	5
		2	Cresta‐Cazis, Planum 2	Plüss	2007	6	2
		2	Cresta‐Cazis, Planum 1	Plüss	2007	8	
		1	Savognin‐Padnal, Horizont E	Bopp‐Ito	Unpub.	6	
		3	Schellenberg‐Borscht 1965	Hartmann‐Frick	1965	14	1
		3	Schellenberg‐Borscht 1937	Kuhn	1937	3	
	Total					73	8
Central	Late Bronze Age	6	Zürich‐Alpenquai	Wettstein	1924	70	39
Plateau	Total					70	39
	Early Bronze Age	5	Meilen‐Obermeilen	Kuhn	1935	26	6
		6	Zürich‐Mozartstrasse, 1o	Hüster Plogmann/Schibler	1997	27	8
		6	Zürich‐Mozartstrasse, 1u	Hüster Plogmann/Schibler	1997	38	7
	Total					91	21
	Late Neolithic	8	Auvernier La Saunerie	Stampfli	1976	56	18
		8	Auvernier Brise‐Lames	Desse	1976	9	
		7	Thielle Wavre, Pont‐de‐Thielle	Chaix	1977	7	4
		6	Zürich‐Mozartstrasse, 2o	Hüster Plogmann/Schibler	1997	44	17
		6	Zürich‐Mozartstrasse, 2u	Hüster Plogmann/Schibler	1997	15	3
		6	Zürich‐Mythenschloss 2.4	Hüster Plogmann/Schibler	1997	11	
		6	Zürich‐Mythenschloss 2.2–3	Hüster Plogmann/Schibler	1997	6	1
		6	Zürich‐Mythenschloss 2.1	Hüster Plogmann/Schibler	1997	2	
		6	Zürich‐Pressehaus C2	Hüster Plogmann/Schibler	1997	4	3
		6	Zürich Seefeld Kan. San. F	Hüster Plogmann/Schibler	1997	1	
		6	Zürich Seefeld Kan. San. E	Hüster Plogmann/Schibler	1997	13	
		6	Zürich Seefeld Kan. San. D	Hüster Plogmann/Schibler	1997	30	8
		6	Zürich Seefeld Kan. San. C/B	Hüster Plogmann/Schibler	1997	17	2
		6	Zürich Seefeld Kan. San. A	Hüster Plogmann/Schibler	1997	9	1
	Total					224	57
					Total	563	143

*Note*. LSI = logarithmic size index; LSI n = number of specimens used for LSI determination; Mc Bd = Metacarpus greatest distal breadth; Mc Bd n = number of specimens providing measurements of the Mc Bd used for sex ratio determination as the effective variable to distinguish the sexes; Unpub. = unpublished; Zürich Seefeld Kan. San. = Zürich Seefeld Kanalisationssanierung.

The samples were grouped by (a) assemblage levels (a site that involved several settlements from different layers and a site that involved a single settlement) and (b) regional levels and by periods (Table S2 and Appendix S3). Sites were separated in different altitudes: Padnal (1,200 m), other Alpine sites (700–800 m), and Plateau sites (400 m). The EBA and MBA Alpine samples were pooled into one period because some Alpine samples were from the transitional period between EBA and MBA (Rageth, 1986). Therefore, the Alpine samples were divided into two groups: (a) EBA and MBA and (b) LBA. Alpine LN samples were too scarce for this study due to insufficient number of settlements in this period, but Plateau LN samples were abundantly available. The Plateau samples were divided into three groups: (a) LN, (b) EBA, and (c) LBA. Plateau MBA sites have not been discovered thus far (Menotti, 2015).

### 2.2 Osteometric samples obtainment and logarithmic size index

The osteometric samples from Padnal were identified as belonging to cattle (Bos taurus) (Bopp‐Ito, unpublished data) following cross‐referencing with published literature (e.g., Schmid, 1972) and the reference collection of the Institute of Integrative Prehistory and Archaeological Science, University of Basel, Switzerland. Samples from Padnal were obtained using metric methods (von den Driesch, 1976) that considered only the completely fused epiphyseal bones, which indicated that the cattle were adults of over 3 years of age, although some subadult cattle have fused epiphyses at two‐and‐a‐half years of age. This inclusion criterion (i.e., adult or subadult) was based on several previous studies (e.g., Becker, 1981; Becker & Johansson, 1981; Habermehl, 1975). Species identification and measurement of the originally reported data from the 32 assemblages were used following a description by Manning et al. (2015) and the references therein.

Calculation of the logarithmic size index (LSI = [log *x* − log *m*] = log (*x*/*m*), where *x* denotes the measurement to be judged and *m* denotes the measurement at the standard reference) is an effective method for estimating body size from specific osteometric parameters obtained from bone fragments, especially for generating a large sample size, and it is widely used in zooarchaeology to study geographical and chronological diversity of animal morphologies (Meadow, 1999; Simpson, Roe, & Lewontin, 1960). The LSI was applied to the osteometric data from Savognin‐Padnal combined with the preexisting data of Swiss LN and Bronze Age cattle following a standardized protocol described by Breuer et al. (1999) and Schibler and Steppan (1999) and the references therein, because most of our samples provided data only on width due to heavy fragmentation. The marginal measurements were excluded following aforementioned protocols and Duval et al. (2013). In total, 563 osteometric samples from postcranial skeletal elements based on eight measurement positions, which were selected to ensure adult or subadult specimens based on fusion of their epiphyses in later stages of age (e.g., Habermehl, 1975), were considered as parameters for the analysis (von den Driesch, 1976; Table 2). Standard reference values for LSI estimation were based on the bone measurements of a 13‐year‐old modern Hinterwälder breed female cow (Z‐2431, withers height approximately 116.9 cm) from the collection of the Institute of Integrative Prehistory and Archaeological Science, University of Basel, as recorded by R. Ebersbach and G. Breuer (Breuer et al., 1999). LSI expresses the size of a cattle specimen relative to that of a reference specimen, with a specimen larger than the reference specimen (LSI value = 0) securing a positive value, and a specimen smaller than the reference specimen securing a negative value (Meadow, 1999). LSIs for each assemblage with at least over 10 observations were compared through notched box plots created using Wessa net software version 1.2.1 (Wessa, 2017), and histograms for Alpine assemblages were generated after Duval et al. (2013) using PAST software version 3.04 (Hammer, Harper, & Ryan, 2001).

**Table 2:** Measurements used to obtain osteometric data from cattle bones for subsequent logarithmic size index analysis (for details see von den Driesch, 1976) and the standard value (Breuer et al., 1999, pp. 227–228)

Skeletal element	Position and standard value (mm)
Humerus	Bp (Greatest proximal breadth), 87.50
Radius	Bd (Greatest distal breadth), 70.60
Ulna	DPA (Depth across the processus anconaeus), 59.24
Femur	DC (Greatest depth of the caput femoris), 45.61
Tibia	Bp (Greatest proximal breadth), 96.71
Metacarpus	Bd (Greatest distal breadth), 55.29
Metatarsus	Bd (Greatest distal breadth), 51.94
Calcaneus	GL (Greatest length), 125.50

The statistical differences between LSIs were examined according to assemblages and periods, following a standardized protocol described by Colominas, Schlumbaum, and Sana (2013) and Duval et al. (2013) and the references therein. The nonparametric Kruskal−Wallis test with one‐way analysis of variance was performed to determine the statistical validity of the differences between the sample medians, followed by a post hoc nonparametric Mann–Whitney *U* test if significant differences were detected. The level of statistical significance was set at *p* = .05. To assess the statistical significance of differences between the all LSIs divided by regions (Alpine and Plateau) and periods, a mixed model (StataCorp, 2013b) with assemblages as random effects was used. Statistical tests for LSI were performed using PAST and STATA software version 13.1 (StataCorp, 2013a).

### 2.3 Sex ratio

Differences in sex ratio possibly cause differences in body size distribution, as male cattle tend to be larger than female cattle. Our samples pooled the data on female, male, and castrated male cattle (Armitage, 1982; Grigson, 1982); however, sex information was not available from all the sites described in the references included in the current study. Therefore, to distinguish the sexes, the greatest distal breadth (Bd) of the metacarpus (see von den Driesch, 1976) as one of the most effective osteometric variable (Davis et al., 2012; Hüster, 1990) was extracted from the osteometric data and used for sex ratio determination (Table 1). Raw osteometric variables (not log‐transformed) were used and analysed by fitting to a finite mixture model (McLachlan & Peel, 2000). The model assumes a mixture of two normal distributions with sex‐specific means and standard deviations. From the model output, the percentage of female cattle can be identified by assuming that the lower mean value corresponds to female cattle. This method provided estimates of the percentage of females (referred to as female probability) and of the sex‐specific means and standard deviations together with 95% confidence intervals. This model is fitted separately for each assemblage with at least six observations.

The variance in the true female probabilities based on the estimated female probabilities was estimated by using a meta‐analytic technique (Russo, 2007). The estimated tau of this variance and the *p* value of a test of the hypothesis that the variance equalled zero are reported. Additionally, a metaregression to evaluate whether female probabilities were associated with the mean LSI in eight assemblages was performed, and *p* < .05 indicated significance. Statistical analyses for sex ratio and its association with the mean LSI were performed using the STATA software version 13.1 (StataCorp, 2013a).

## 3 RESULTS

### 3.1 Sex ratio diversity of cattle between assemblages and periods

**Figure 2:**
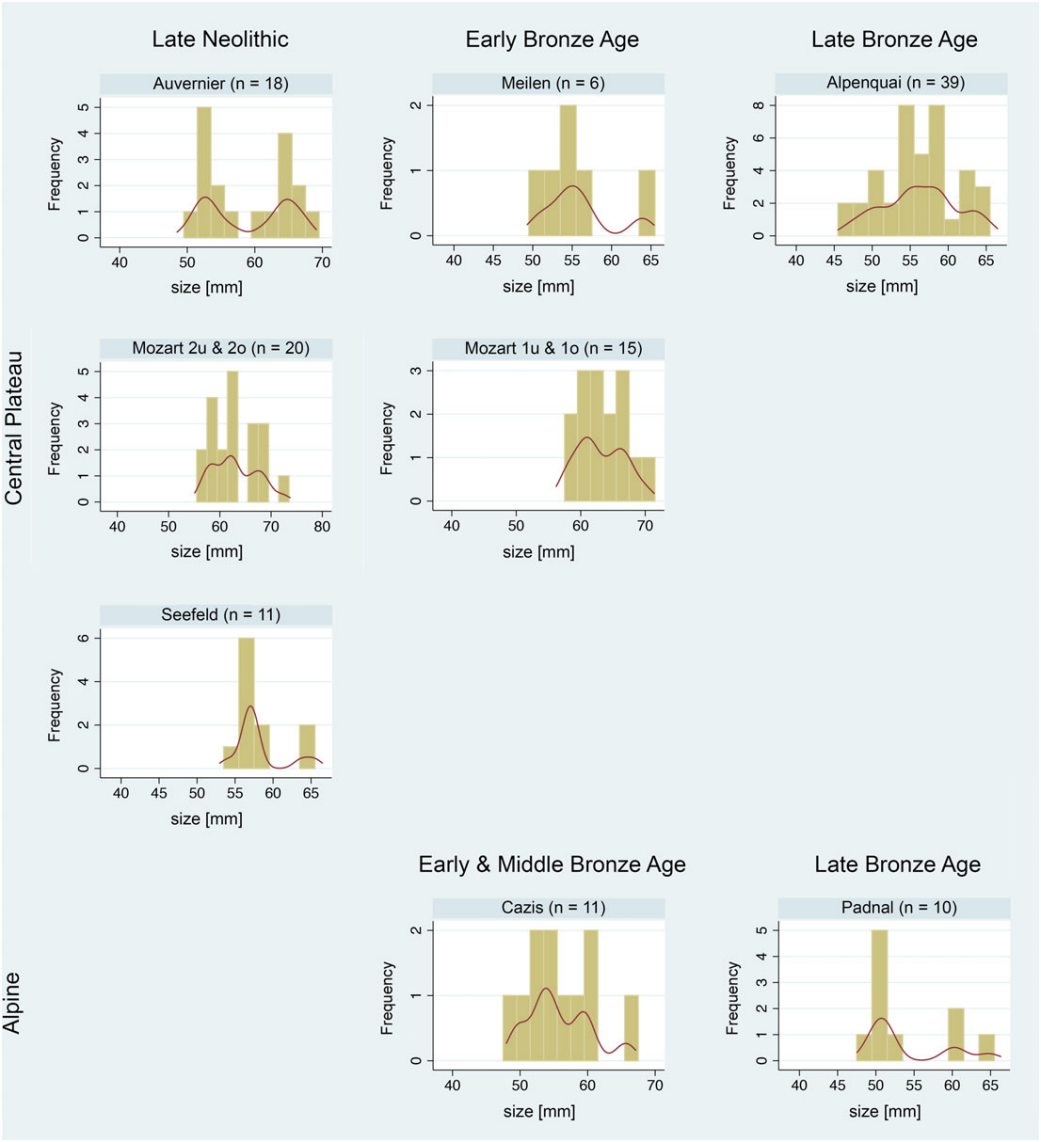
Histograms with Kernel density curves showing the distribution of the greatest distal breadth (Bd) measurements of cattle metacarpus from the Late Neolithic and Bronze Age assemblages. These data were used in a finite mixture model. For site names and details, see Table 1 [Colour figure can be viewed at http://wileyonlinelibrary.com]

**Figure 3:**
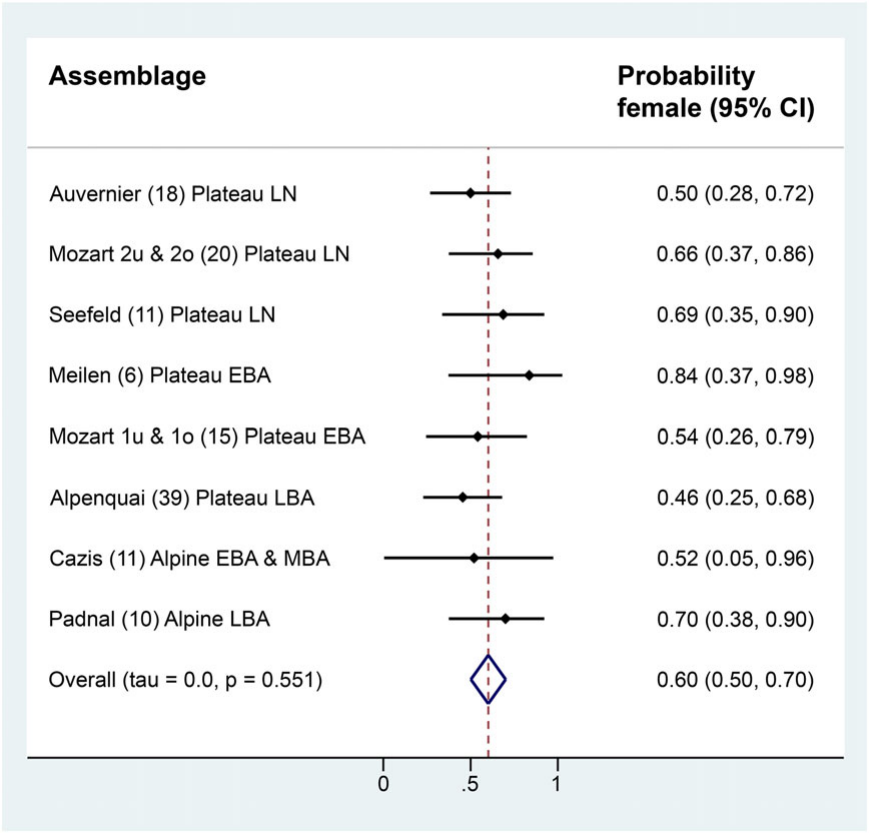
Forest plots of the probable proportions of female cattle in the Alpine and Central Plateau assemblages. Assemblages providing more than six measurements of the greatest distal breadth (Bd) of cattle metacarpus were included. Numbers in parentheses next to the assemblage names indicate the sample sizes. Cl = confidence interval; EBA = Early Bronze Age; LBA = Late Bronze Age; LN = Late Neolithic; MBA = Middle Bronze Age; Plateau = Central Plateau. For site names and details, see Table 1 [Colour figure can be viewed at http://wileyonlinelibrary.com]

The distribution of metacarpus Bd measurements of cattle bones from assemblages, with at least six observations, revealed a clear bimodal sex distribution (Figure 2). Hence, it is possible to fit a finite mixture model successfully for each settlement. All estimates are reported in Table S3. No evidence for a variation in true sex ratios over time or within regions was found (tau = 0.0; *p* = .55), and the average female probability across assemblages was 60% (Figure 3). Furthermore, no evidence for an association between the mean LSI and female probability was found (*p* > .05).

### 3.2 Size diversity of cattle between assemblages

**Figure 4:**
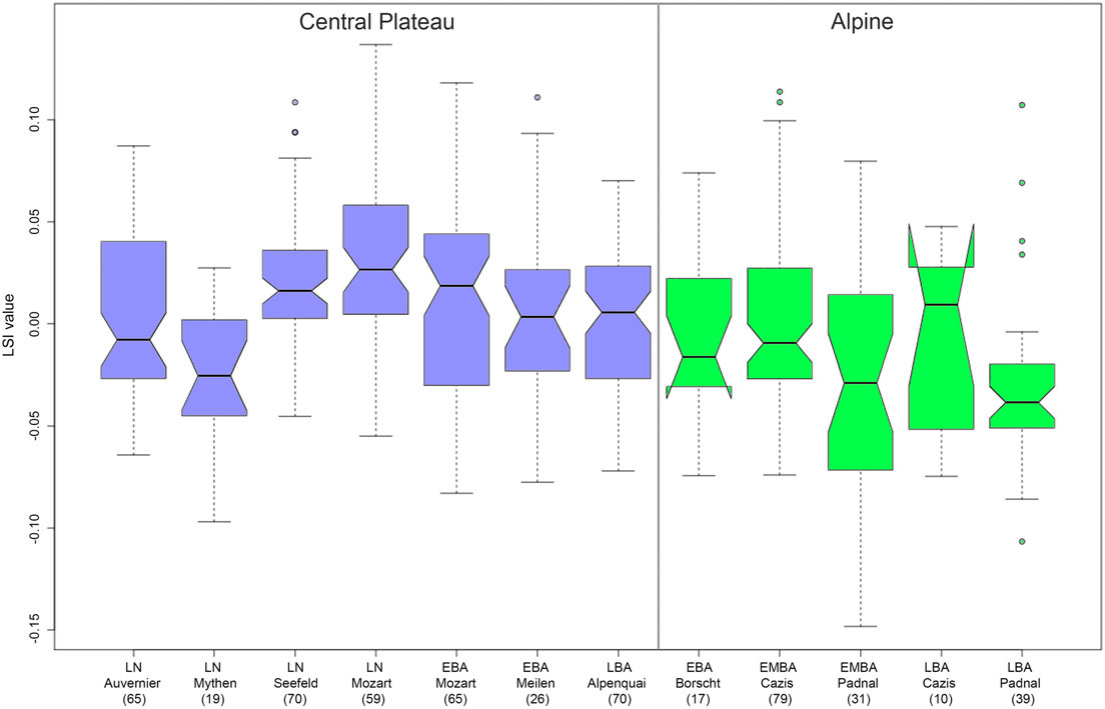
The logarithmic size index (LSI) of cattle in the Alpine and Central Plateau assemblages between the Late Neolithic and Bronze Age periods. Numbers in parentheses indicate the sample size used to obtain LSI. EBA = Early Bronze Age; EMBA = Early and Middle Bronze Age; LBA = Late Bronze Age; LN = Late Neolithic. For site names and details, see Table 1 [Colour figure can be viewed at http://wileyonlinelibrary.com]

**Figure 5:**
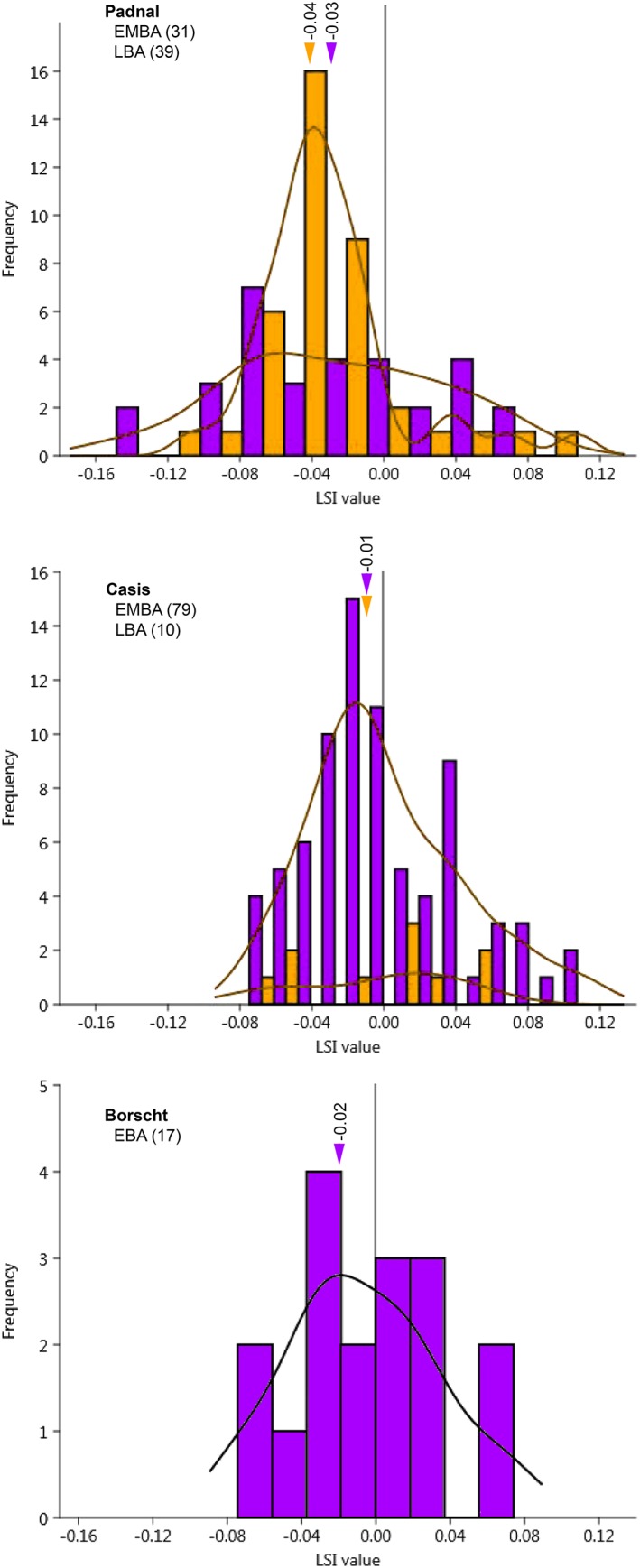
Histograms of the logarithmic size index (LSI) with Kernel density curves of cattle from the Alpine Bronze Age assemblages. EBA and EMBA are dark gray (purple) and LBA is light gray (orange) colour. Numbers in parentheses indicate the sample size used to obtain LSI. The arrows indicate the median value for each assemblage (after Duval et al., 2013). EBA = Early Bronze Age; EMBA = Early and Middle Bronze Age; LBA = Late Bronze Age. For site names and details, see Table 1 [Colour figure can be viewed at http://wileyonlinelibrary.com]

**Figure 6:**
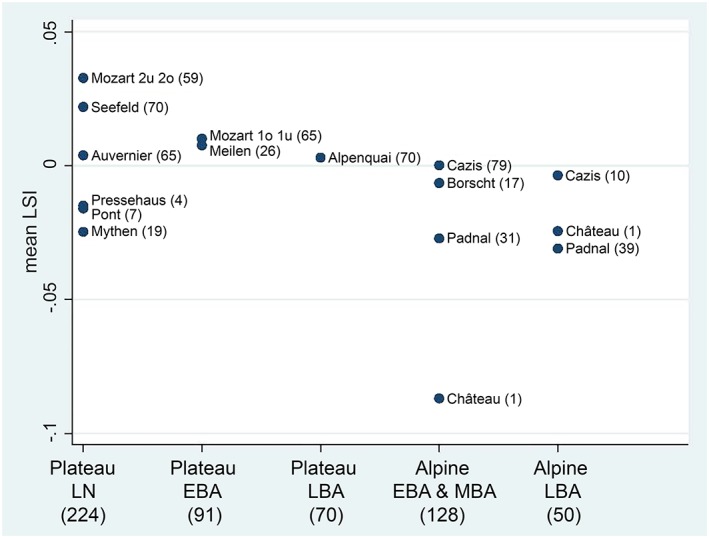
Mean of the logarithmic size index (LSI) of cattle in the Alpine and Central Plateau assemblages between the Late Neolithic and Bronze Age periods used to a meta‐regression to evaluate whether means were associated with female probabilities. Numbers in parentheses indicate the sample sizes used to obtain LSI. EBA = Early Bronze Age; LBA = Late Bronze Age; LN = Late Neolithic; MBA = Middle Bronze Age; Plateau = Central Plateau. For site names and details, see Table 1 [Colour figure can be viewed at http://wileyonlinelibrary.com]

The LSI values with over 10 observations revealed a highly significant difference in size between the cattle populations divided by assemblages and periods (*χ* ^*2*^ = 82.7, *p* < .0001). The size of Plateau cattle, except Zürich‐Mythenschloss cattle, was homogenized. However, the Schellenberg‐Borscht and Cresta‐Cazis (hereafter calls Borscht and Cazis) Alpine cattle were larger than those at Padnal, which were the smallest (Figure 4). Pairwise comparisons confirmed that Padnal cattle were significantly smaller (Table 3). However, the histograms of all LSIs from Alpine assemblages showed that there was hardly any size reduction in Alpine cattle inside the settlements, that is, in Cazis and Padnal (Figure 5). The distribution of mean LSI (Figure 6) roughly grouped cattle populations into three sizes: Plateau, Borscht/Cazis, and Padnal cattle. Statistical parameters are summarised in Table S4.

**Table 3:** Mann–Whitney *U* test pairwise comparisons of the logarithmic size index (LSI) of cattle in the Alpine and Central Plateau assemblages between the Late Neolithic and Bronze Age periods. Numbers in parentheses indicate the sample size used to obtain LSI. For site names and details, see Table 1

Period	Late Neolithic				Bronze Age							
Region	Central Plateau							Alpine				
	LN	LN	LN	LN	EBA	EBA	LBA	EBA	EMBA	EMBA	LBA	LBA
Assemblage	Auver.	Mythen.	Seefeld	Mozart	Mozart	Meilen	Alpenq.	Borscht	Cazis	Padnal	Cazis	Padnal
Sample n	65	19	70	59	65	26	70	17	79	31	10	39
LN Auvernier		**<.05**	**<.01**	**<.001**	0.4232	0.6477	0.9543	0.4232	0.7254	**<.05**	0.7025	**<.0001**
LN Mythen			**<.0001**	**<.0001**	**<.01**	**<.05**	**<.01**	0.2050	**<.05**	0.7796	0.1033	0.2021
LN Seefeld				0.1187	0.2327	0.0590	**<.05**	**<.01**	**<.001**	**<.0001**	0.1437	**<.0001**
LN Mozart					**<.05**	**<.05**	**<.001**	**<.01**	**<.0001**	**<.0001**	**<.05**	**<.0001**
EBA Mozart						0.6637	0.3326	0.1716	0.1996	**<.01**	0.4088	**<.0001**
EBA Obermeilen							0.8755	0.3646	0.4078	**<.05**	0.7911	**<.001**
LBA Alpenquai								0.3683	0.3340	**<.01**	0.5852	**<.0001**
EBA Borscht									0.6110	0.2274	0.7441	**<.05**
EMBA Cazis										**<.05**	0.9224	**<.0001**
EMBA Padnal											0.2425	0.8039
LBA Cazis												0.0847
LBA Padnal												

Significant p values are in bold. α = .05.

*Note*. α = significance level; Alpenq. = Alpenquai; Auver. = Auvernier; EBA = Early Bronze Age; EMBA = Early and Middle Bronze Age; LBA = Late Bronze; LN = Late Neolithic; MBA = Middle Bronze Age; Mythen. = Mythenschloss.

### 3.3 Size diversity of cattle between regions

**Figure 7:**
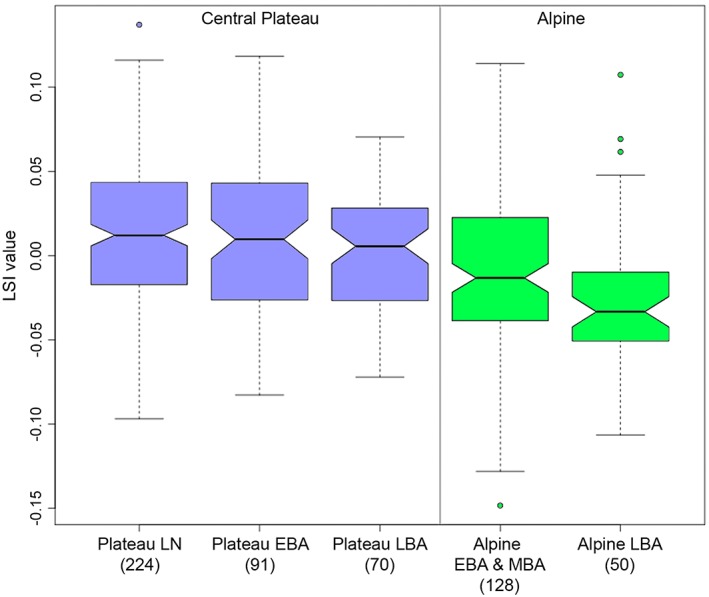
The logarithmic size index (LSI) of cattle in the Alpine and Central Plateau regions between the Late Neolithic and Bronze Age periods. Numbers in parentheses indicate the sample sizes used to obtain LSI. EBA = Early Bronze Age; LBA = Late Bronze Age; LN = Late Neolithic; MBA = Middle Bronze Age [Colour figure can be viewed at http://wileyonlinelibrary.com]

All pooled LSI data divided by regions and periods showed that size distributions varied widely among the Alpine and Plateau cattle populations (Figure 7). Alpine cattle showed a marked decrease in the median LSI value with time, in contrast to no changes in Plateau cattle LSI values. However, this may have been due to the slight contribution of the Cazis LBA cattle, owing to the small sample size, as indicated in Figure 4. A mixed model analysis revealed that even after taking into account the settlements as factors of their own, the difference in size between the Alpine and Plateau cattle was significant (*p* = .033). Evidence for a time trend was not found (*p* = .610), even inside the settlements, as evident from Figures 5 and 6. Statistical parameters are summarised in Table S5.

## 4 DISCUSSION

It is evident that Alpine cattle tended to be smaller than Plateau cattle, but there was no evidence for any time trends. In general, a substantial variation in size distribution across settlements makes the assessment of global trends difficult. However, cattle from Padnal was relatively small throughout the Bronze Age. Our study indicates that comparing samples at the assemblage level is important for a better understanding of cattle size distributions, because it seems necessary to take the archaeological circumstances of each individual site, including its distinct location, environment, economy, and links with cattle husbandry, such as the identified number or the mortality profile, into consideration. Several potential factors may have contributed to the diversity in cattle size distribution, as discussed below.

As there was no evidence for variations in sex ratios between assemblages, this factor can be excluded from the list of potential explanations. The 60% female probability across assemblages might be due to samples belonging to mature individuals. Hence, there was sexual selection of offspring; mostly males were killed for meat and females were retained for secondary products (Halstead & Isaakidou, 2011). The LN Zürich‐Mythenschloss small cattle might have been mostly female due to the high mortality profile of offspring calves (Hüster Plogmann & Schibler, 1997). The correlation of sex ratios with mean LSI from a corresponding metaregression also did not hint at an influence of sex ratios on size distribution. Therefore, our hypothesis that the intensification of dairy production based on the increase in senile cattle at the Alpine sites during the LBA was not supported. The Alpine cattle, especially Padnal cattle, could have been exploited for meat and as working power in agriculture and bronze production (Bopp‐Ito, 2012; Plüss, 2011; Stopp, 2015). However, a larger sample size is necessary to draw definite conclusions regarding the role of sex ratios in determining size diversity.

Although there was no time trend, significant trends were found between the two regions and 12 assemblages. Cattle size distributions seemed to be related to the altitude for large Plateau cattle in the flat Plateau, middle Borscht and Cazis cattle in the lower Alps, and small Padnal cattle in the middle of the Alps. These habitats were formed by various types of fodder in soil, temperature, or agropastoral land use (Davis, 1981; Duval et al., 2013; Jacomet, 1998; Knockaert et al., 2017; Reitmaier, 2012), and the quality of diet could also have affected cattle size (Breuer et al., 1999). However, the lack of data based on various proxies, for example, pollen or tree rings, that enable dating and climate reconstruction, makes it difficult to explore the climatic effects on the samples from Alpine sites (Schibler, 2017).

As to the reason why small Padnal cattle suddenly appeared in the Alps, there is a possibility that they could be an introduced allochthonous population (Gaastra, 2014; MacKinnon, 2010), but it is difficult to discuss this due to the lack of the osteometric samples from Neolithic Alpine cattle. There is an alternative possibility that they could be a migrated population accompanied by migrants (Grupe et al., 2017) from areas outside the Swiss Alpine region, such as from the southern parts of the Alps (northern Italy and Tyrolian Austria), because the size of cattle in the southern parts of the Alps over 1,000 m above the sea level (e.g., Riedel & Tecchiati, 1998) is smaller than that in the northern parts (Riedel & Tecchiati, 2001; Stopp, 2015; Trixl et al., 2017), but homogeneous with that of Padnal cattle (Bopp‐Ito, unpublished data), indicating a similar environment and close economic relationship between Padnal and the southern communities.

The location of Padnal beside the road that crosses over the Julier pass towards northern Italy, and excavation of outstanding foreign objects, particularly those with attributes of northern Italy (Rageth, 1986), suggest a deep relationship with north Italian communities through traffic and trade networks (Della Casa, 2007; Jecker, 2015; Jennings, 2015). The southern Alpine people who might have had a technology of bronze production (Marti‐Grädel, Stopp, Deschler‐Erb, Hüster Plogmann, & Schibler, 2012) perhaps migrated into the northern parts of the Alps with their livestock and selected the location at Padnal to build their settlement for bronze production (Della Casa et al., 2016; Rageth, 1986). The lack of change in the size of cattle in each Alpine site might reflect their stable animal management strategies throughout the Bronze Age. Nevertheless, the limited number of sites with animal remains located close to the southern Alpine communities, making it difficult to conclude whether small cattle at Padnal were an exception or occurred commonly. In contrast, the extra‐large Near East mitochondrial d‐loop haplogroup T2 cattle (Bonfiglio et al., 2010), to date have been found only from the Roman site in Switzerland (Schlumbaum, Turgay, & Schibler, 2006), were excavated from a house with objects with south German attributes in the EBA Padnal settlement (Horizont E) (Bopp‐Ito, 2012), which suggests the migration of large cattle from north routes and small cattle from south routes. However, it is difficult to confirm this possibility without genetic (e.g., Schibler & Schlumbaum, 2007), geometric morphometrics (Cucchi et al., 2016), isotopic, or strontium (Grupe et al., 2017; Reitmaier et al., 2017) evidence to reconstruct phylogenetic relationships and places of origin and migration.

The middle‐sized Alpine cattle at Borscht and Cazis might have migrated from the southern but more so from the northern communities, such as Plateau or South Germany, and these cattle might have been phylogenetically related because these sites possessed objects with northern attributes much more than Padnal during the EBA (Jecker, 2015). As they were located along the river Rhine (cf. Figure 1), contact with the northern communities through the river and lakes was more probable and frequent than with Padnal, which was located in the higher mountains. The different strength of economic interrelationships between Alpine and other geographically closed communities might have resulted in three sizes of Swiss Bronze Age cattle. The migrated Alpine cattle were well adapted to the environment at different altitudes, increased their populations, and did not change their size in each settlement, in spite of the change of culture and cattle husbandry practices, such as change of cattle exploitation during the LBA. Further research with large sample sizes and the aforementioned interdisciplinary approaches are necessary for verifying our hypotheses.

## 5 CONCLUSION

Osteometric data of Swiss LN and Bronze Age cattle from different regions and assemblages across periods were used to evaluate the differences in the size of cattle using the LSI and to explore the effect of sex ratio on size, based on a finite mixture model and a meta‐analytic technique using the measurements of the greatest Bd of the metacarpus. Sex ratio did not correlate with mean LSI. Our hypothesis that the increase in female cattle for dairy production in the LBA Alpine sites was not proved. There was no evidence for time trends but there were differences in size between cattle populations that were presumably caused by varied nutrition in the diet linked to the altitude, but this is open to debate. The smallest Padnal cattle might have been migrated populations accompanying migrants mainly from the southern parts of the Alps, and they could have been exploited for meat and as working power for bronze production, mining, and agriculture. Middle‐sized Borscht and Cazis cattle might be more phylogenetically related to large Plateau cattle because these sites might have been economically interrelated more frequently with northern communities. Further investigations with larger sample sizes and interdisciplinary studies, including genetics and geometric morphometrics to show a possible link between cattle populations, isotopic analyses to show diet variations, and strontium analyses to show the cattle and human migration, are required to confirm this possibility.

## FUNDING

This research did not receive any specific grant from funding agencies in the public, commercial, or not‐for‐profit sectors.

## CONFLICTS OF INTEREST

The authors declare that they have no conflict of interest.

## Supporting information

Figure S1. Cattle proportion.
Figure S2. Cattle mortality profile.
Table S1. Dating of the assemblages.
Table S2. Detailed list of the studied sites.
Table S3. Statistical summary for Figure 2 and 3.
Table S4. Statistical summary for Figure 4.
Table S5. Statistical summary for Figure 7.
Appendix S1. References for Table 1.
Appendix S2. References for Table S1.
Appendix S3. References for Table S2.
