# Supplementary material for: Size diversity in Swiss Bronze Age cattle
Source: Int J Osteoarchaeol. 2018 Apr 17;28(3):294–304. doi: 10.1002/oa.2654 (PMC6032853; doi:10.1002/oa.2654)
Supplement: Supplementary file 1 — Figure S1. Cattle proportion. Figure S2. Cattle mortality profile. Table S1. Dating of the assemblages. Table S2. Detailed list of the studied sites. Table S3. Statistical summary for Figure 2 and 3. Table S4. Statistical summary for Figure 4. Table S5. Statistical summary for Figure 7. Appendix S1. References for Table 1. Appendix S2. References for Table S1. Appendix S3. References for Table S2. [file OA-28-294-s001.zip › Bopp-Ito_et al_Supplementary Material.docx]

**Figure S1.** Proportion of the number of identified specimens (NISP) of cattle in each assemblage. Numbers in parentheses indicate the NISP of cattle. For site names and details, see Table 1.

**
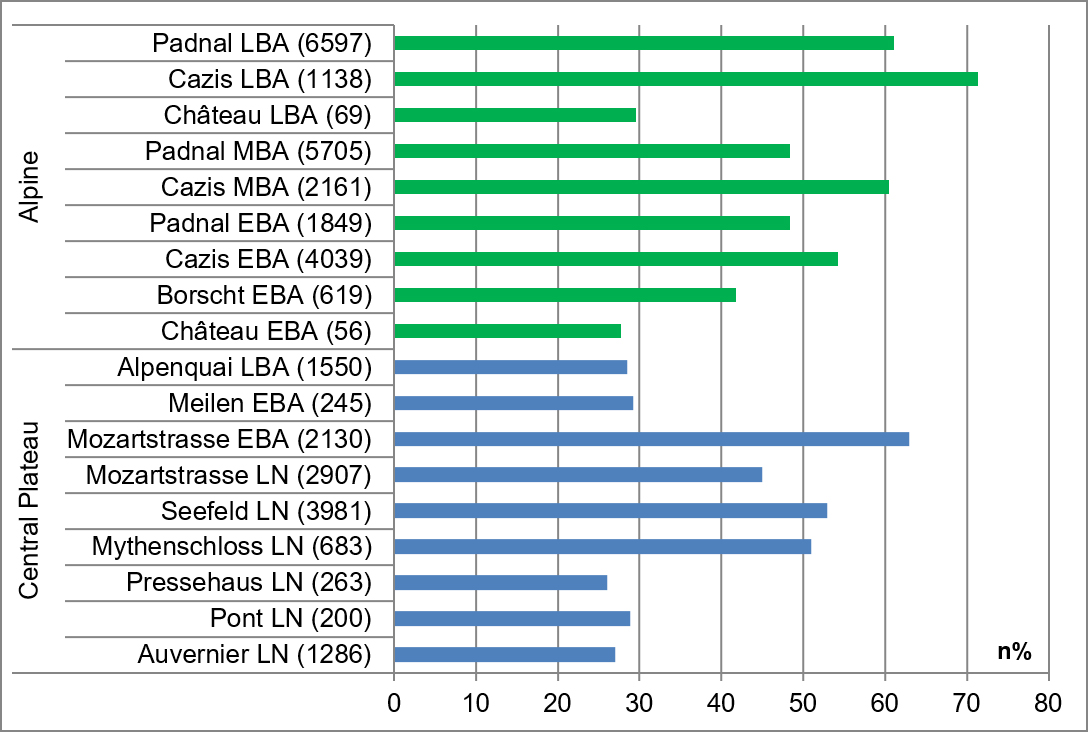
**

*LN = Late Neolithic; EBA = Early Bronze Age; MBA = Middle Bronze Age; LBA = Late Bronze Age***.**

**Figure S2.** Cattle mortality profile based on tooth eruption and wear from the settlements in Zurich area and Savognin-Padnal. Numbers in parentheses indicate the number of teeth used to obtain the mortality profile. For site names and details, see Table 1.

*LN = Late Neolithic; EBA = Early Bronze Age; MBA = Middle Bronze Age; LBA = Late Bronze Age; Mythen = Mythenschloss; Presse. = Pressehaus; Seefeld Kan. San. = Seefeld Kanalisationssanierung; Mozart = Mozartstrasse; Padnal = Savognin-Padnal.*

**Table S1.** Dating of the assemblages listed in Table 1 corresponding to the locations shown in Figure 1.

| **Region** | **Period** | **Map**  **No.** | **Assemblage** | **Method** | **Dating BC** | **Dating Reference** |
| --- | --- | --- | --- | --- | --- | --- |
| **Alpine** | **LBA** | 4 | Ayent Le Château | C14 | 1210-906 | David-El Biali M, 1990 |
|  |  | 2 | Cresta-Cazis, N-Planum 14 | C14  Typology | 1350-1100/800  LBA-Iron Age 1300-400 | Wyss R, 2002  Murbach-Wende I, 2016 |
|  |  | 1 | Savognin-Padnal, Horizont B | C14 | 1350/1300-900/800 | Rageth J, 1986 & pers. Com. |
|  |  |  |  |  |  |  |
|  | **MBA** | 2 | Cresta-Cazis, N-Planum 10-12 | C14  C14 | 1800-1550  1750-1300 | Wyss R, 2002  Murbach-Wende I, 2016 |
|  |  | 1 | Savognin-Padnal, Horizont C | C14 | 1450-1350/1300 | Rageth J, 1986 & pers. Com. |
|  |  | 1 | Savognin-Padnal, Horizont D | C14 | 1550-1450 | Rageth J, 1986 & pers. Com. |
|  |  |  |  |  |  |  |
|  | **EBA** | 4 | Ayent Le Château | Typology | BzA3-BzA4 | David-El Biali M, 1990 |
|  |  | 2 | Cresta-Cazis, N-Planum 1-5, 8  Cresta-Cazis, N-Planum 8  Cresta-Cazis, N-Planum 1-5 | C14  C14  C14 | 2400-1800  1850-1700  2000-1850 | Wyss R, 2002  Murbach-Wende I, 2016  Murbach-Wende I, 2016 |
|  |  | 1 | Savognin-Padnal, Horizont E | C14 | 1950/1900-1550 | Rageth J, 1986 & pers. Com. |
|  |  | 3 | Schellenberg-Borscht 1937, 1965 | Typology | Early Bronze Age 2000 | Hild A, 1935, 1936 |
|  |  |  |  |  |  |  |
| **Plateau** | **LBA** | 6 | Zürich-Alpenquai | Typology | Late Bronze Age | Hochuli S et al., 1998 (SPM III) |
|  |  |  |  |  |  |  |
|  | **EBA** | 5 | Meilen-Obermeilen | Typology | Early Bronze Age | Kuhn M, 1935, Keller F, 1870, Hochuli S et al., 1998 (SPM III) |
|  |  | 6 | Zürich-Mozartstrasse, 1u, 1o | Typology | Early Bronze Age | Bleicher N, 2011 |
|  |  |  |  |  |  |  |
|  | **LN** | 8 | Auvernier La Saunerie | Den. | 2800-2450 | Stöckli E et al., 1995 (SPM II) |
|  |  | 8 | Auvernier Brise-Lames | Den. | 2800-2450 | Stöckli E et al., 1995 (SPM II) |
|  |  | 7 | Thielle Wavre, Pont-de-Thielle | Den. | 2701 | Stöckli E et al., 1995 (SPM II) |
|  |  | 6 | Zürich-Mozartstrasse, 2o | Den. | 2605-2568 | Gross-Klee E, 1997 |
|  |  | 6 | Zürich-Mozartstrasse, 2u | Den. | 2625-2605 | Gross-Klee E, 1997 |
|  |  | 6 | Zürich-Mythenschloss 2.1 | Den. | 2548 | Stöckli E et al., 1995 (SPM II) |
|  |  | 6 | Zürich-Mythenschloss 2.2, 2.3, 2.4 | Den. | 2680 | Stöckli E et al., 1995 (SPM II) |
|  |  | 6 | Zürich-Pressehaus C2 | Den. | 2719-2713, 2690-2683 | Stöckli E et al., 1995 (SPM II) |
|  |  | 6 | Zürich Seefeld Kan. San. A, C/B, D, E, F | Den. | 2718-2675 | Gross-Klee E, 1997 |

*Den. = Dendrochronology. Pers. com. = Personal communication; Bz = Bronzezeit (Bronze Age); LN = Late Neolithic; EBA = Early Bronze Age; MBA = Middle Bronze Age; LBA = Late Bronze Age; Zürich Seefeld Kan. San. = Zürich Seefeld Kanalisationssanierung.*

**Table S2.** Detailed list of the studied sites. The map number (Map No.) corresponds to the locations shown in Figure 1. For details of site names and references, see Table 1.

| **Map** | **Site** | **Grouping** | **Topography** | **Altitude** | **Location** | **Culture** | | | |
| --- | --- | --- | --- | --- | --- | --- | --- | --- | --- |
| **No.** |  |  |  | **(*ca*. m)** | **near (by)** | **LN** | **EBA** | **MBA** | **LBA** |
| 1 | Padnal | Alpine EMBA, LBA | Middle Alps | 1223 | Julier pass |  | Inneralpine | Inneralpine | Mix of RSFO, Main-Schwaben, Laugen-Melaun |
| 2 | Cazis | Alpine EMBA, LBA | Lower Alps | 765 | River Rhine |  | Inneralpine | Inneralpine | Mix of RSFO, Main-Schwaben, Laugen-Melaun |
| 3 | Borscht | Alpine EMBA | Lower Alps | 690 | River Rhine |  | Inneralpine | Inneralpine | Mix of RSFO, Main-Schwaben, Laugen-Melaun |
| 4 | Château | Alpine EMBA, LBA | Lower Alps | 840 | River Rhone |  | Rhone |  | RSFO |
| 6 | Alpenquai | Plateau LBA | East Plateau | 400 | Lake Zurich |  |  |  | RSFO |
| 5 | Meilen | Plateau EBA | East Plateau | 404 | Lake Zurich |  | Arbon |  |  |
| 6 | Mozartstrasse | Plateau LN, EBA | East Plateau | 404 | Lake Zurich | Schnurkeramik, Glockenbecher | Arbon |  |  |
| 6 | Mythenschloss | Plateau LN | East Plateau | 404 | Lake Zurich | Schnurkeramik, Glockenbecher |  |  |  |
| 6 | Pressehaus | Plateau LN | East Plateau | 404 | Lake Zurich | Schnurkeramik, Glockenbecher |  |  |  |
| 6 | Seefeld | Plateau LN | East Plateau | 404 | Lake Zurich | Schnurkeramik, Glockenbecher |  |  |  |
| 7 | Pont | Plateau LN | West Plateau | 430 | Lake Neuchâtel | Schnurkeramik, Glockenbecher |  |  |  |
| 8 | Auvernier | Plateau LN | West Plateau | 430 | Lake Neuchâtel | Schnurkeramik, Glockenbecher |  |  |  |
| *LN = Late Neolithic; EBA = Early Bronze Age; EMBA = Early and Middle Bronze Age; LBA = Late Bronze Age; RSFO = Rhein-Schweiz-Ostfrankreich-Kultur.* | | | | | | | | | |

**Table S3.** Results of a *finite mixture mode*l analysis in Figure 2 and 3. The greatest distal breadth (Bd) measurements of cattle metacarpus were used in the models.

| **Region** | **Period** | **Assemblage** | **Data**  **(n)** | **Probability female**  **(95% CI)** | **Mean [mm] female**  **(95% CI)** | **Mean [mm] male & cast.**  **(95% CI)** | **SD**  **female**  **(95% CI)** | **SD**  **male & cast.**  **(95% CI)** |
| --- | --- | --- | --- | --- | --- | --- | --- | --- |
| Alpine | LBA | Savognin-Padnal,  Horizont B | 10 | 0.700  (0.376, 0.900) | 50.7  (50.0, 51.3) | 61.8  (59.3, 64.3) | 0.83  (0.49, 1.40) | 2.20  (0.99, 4.89) |
|  | EBA &  MBA | Cresta-Cazis,  Planum 1-5, 8, 10-12 | 11 | 0.520  (0.051, 0.957) | 52.6  (50.0, 55.2) | 59.3  (50.6, 68.0) | 2.21  (1.00, 4.91) | 3.88  (1.12, 13.43) |
| Central Plateau | LBA | Zürich-Alpenquai | 39 | 0.455  (0.252, 0.675) | 52.6  (51.0, 54.2) | 59.3  (57.7, 60.9) | 3.48  (2.12, 5.71) | 3.51  (2.23, 5.54) |
|  | EBA | Meilen-Obermeilen | 6 | 0.836  (0.370, 0.978) | 53.6  (53.6, 53.6) | 63.9  (63.9, 63.9) | 2.19  (1.18, 4.06) | 0.00  (0.00, 0.00) |
|  | EBA | Zürich-Mozartstrasse,  1u, 1o | 15 | 0.541  (0.264, 0.794) | 60.2  (58.7, 61.7) | 66.5  (64.6, 68.3) | 1.69  (0.85, 3.35) | 1.92  (0.93, 3.96) |
|  | LN | Auvernier La Saunerie | 18 | 0.500  (0.284, 0.716) | 53.1  (51.9, 54.4) | 64.7  (63.5, 66.0) | 1.91  (1.20, 3.05) | 1.93  (1.21, 3.09) |
|  | LN | Zürich-Mozartstrasse,  2u, 2o | 20 | 0.657  (0.374, 0.860) | 60.4  (58.7, 62.1) | 68.0  (65.6, 70.4) | 2.44  (1.43, 4.16) | 2.21  (0.96, 5.13) |
|  | LN | Zürich Seefeld Kan San.  A, C/B, D, E, F | 11 | 0.687  (0.347, 0.900) | 57.0  (56.7, 57.4) | 60.5  (55.1, 65.9) | 0.46  (0.28, 0.77) | 4.91  (2.31, 10.44) |

*LN = Late Neolithic; EBA = Early Bronze Age; MBA = Middle Bronze Age; LBA = Late Bronze Age; Zürich Seefeld Kan. San. = Zürich Seefeld Kanalisationssanierung; CI = confidence interval; SD = standard deviation; Male & cast. = male and castrated male cattle.*

**Table S4.** Summary of the descriptive statistics of the osteometric data used in Figure 4.

| **Period** | **Late Neolithic** | | | | | | **Bronze Age** | | | | | | | |
| --- | --- | --- | --- | --- | --- | --- | --- | --- | --- | --- | --- | --- | --- | --- |
| **Region** | **Central Plateau** | | | | | | | | | **Alpine** | | | | |
| **Settlement** | LN | LN | LN | LN | LN | LN | EBA | EBA | LBA | EBA | EMBA | EMBA | LBA | LBA |
|  | Auver. | Pont | Mythen | Presse | Seefeld | Mozart | Mozart | Meilen | Alpenq. | Borscht | Cazis | Padnal | Cazis | Padnal |
| N | 65 | 7 | 19 | 4 | 70 | 59 | 65 | 26 | 70 | 17 | 79 | 31 | 10 | 39 |
| Min. | -0.064 | -0.052 | -0.097 | -0.086 | -0.045 | -0.055 | -0.083 | -0.077 | -0.072 | -0.074 | -0.074 | -0.148 | -0.075 | -0.107 |
| Max. | 0.087 | 0.040 | 0.027 | 0.017 | 0.109 | 0.137 | 0.118 | 0.111 | 0.070 | 0.074 | 0.114 | 0.080 | 0.048 | 0.107 |
| Mean | 0.004 | -0.016 | -0.025 | -0.015 | 0.022 | 0.033 | 0.010 | 0.008 | 0.003 | -0.006 | 0.000 | -0.027 | -0.004 | -0.031 |
| Std. error | 0.005 | 0.012 | 0.007 | 0.024 | 0.004 | 0.006 | 0.006 | 0.009 | 0.005 | 0.010 | 0.005 | 0.010 | 0.014 | 0.006 |
| Std. dev. | 0.042 | 0.033 | 0.032 | 0.049 | 0.032 | 0.043 | 0.049 | 0.047 | 0.038 | 0.040 | 0.043 | 0.058 | 0.043 | 0.040 |
| Median | -0.008 | -0.034 | -0.025 | 0.005 | 0.016 | 0.026 | 0.019 | 0.003 | 0.006 | -0.016 | -0.010 | -0.029 | 0.009 | -0.038 |

N = number of osteometric samples; Min. = Minimum; Max. = Maximum; Std. error = Standard error; Std. dev. = Standard deviation. LN = Late Neolithic; EBA = Early Bronze Age; EMBA = Early and Middle Bronze Age; LBA = Late Bronze Age. Auver. = Auvernier sites; Pont = Pont-de-Thielle; Mythen. = Mythenschloss; Presse. = Pressehaus; Mozart = Mozartstrasse; Meilen = Obermeilen; Alpenq. = Alpenquai. For settlement names and details, see Table 1.

**Table S5.** Summary of the descriptive statistics of the osteometric data used in Figure 7.

|  | Plateau  LN | Plateau EBA | Plateau  LBA | Alpine EMBA | Alpine  LBA |
| --- | --- | --- | --- | --- | --- |
| N | 224 | 91 | 70 | 128 | 50 |
| Min. | -0.097 | -0.083 | -0.072 | -0.148 | -0.107 |
| Max. | 0.137 | 0.118 | 0.070 | 0.114 | 0.107 |
| Mean | 0.014 | 0.009 | 0.003 | -0.008 | -0.025 |
| Std. error | 0.003 | 0.005 | 0.005 | 0.004 | 0.006 |
| Std. dev. | 0.042 | 0.048 | 0.038 | 0.048 | 0.041 |
| Median | 0.012 | 0.010 | 0.006 | -0.013 | -0.033 |

*N = number of osteometric samples; Min. = Minimum; Max. = Maximum; Std. error = Standard error; Std. dev. = Standard deviation. Plateau = Central Plateau; LN = Late Neolithic; EBA = Early Bronze Age; EMBA = Early and Middle Bronze Age; LBA = Late Bronze Age.*

**Appendix S1**. References used for studying the animal bones listed in Table 1.

Chaix L. 1977. La faune de la station de Pont-de-Thielle (néolithique récent). *Archives scientifiques* **30(3)** : 433−464.

Chaix L. 1990. La faune d’Ayent-le-Château (Valais, Suisse; Bronze ancien et Bronze final). *Jahrbuch der Schweizerischen Gesellschaft für Ur- und Frühgeschichte* 73, Huber & Co: Frauenfeld; 44−46.

Desse J. 1976. *La faune du site archéologique Auvernier-Brise Lames, canton de Neuchâtel (Suisse)*. Thèse, Université Poitiers: Neuchâtel.

Hartmann-Frick H. 1965. Die Fauna der befestigten Höhensiedlung auf dem Borscht, Fürstentum Liechtenstein. *Jahrbuch des Historischen Vereins für das Fürstentum Liechtenstein* **63**:187−253.

Hüster Plogmann H, Schibler J. 1997. Archäozoologie. In *Ökonomie und Ökologie neolithischer und bronzezeitlicher Ufersiedlungen am Zürichsee, Ergebnisse der Ausgrabungen Mozartstrasse, Kanalisationssanierung Seefeld, AKAD/Pressehaus und Mythenschloss in Zürich*, Schibler J, Hüster Plogmann H, Jacomet S, Brombacher C, Gross-Klee E, Rast-Eicher A (eds.). Monographien der Kantonsarchäologie Zürich 20: Zürich und Egg; 40−121.

Kuhn E. 1935. Die Fauna des Pfahlbaues Obermeilen am Zürichsee. *Vierteljahrsschrift der Naturforschenden Gesellschaft in Zürich* **80**: 65−154.

Kuhn E. 1937. Die Fauna der Wallsiedlung im Borscht (Endneolithikum-La Tène). *Jahrbuch des Historischen Vereins für das Fürstentum Liechtenstein* **37**: 4−42.

Plüss P. 2007. *Archäozoologische Untersuchungen der Tierknochen aus Cresta-Cazis (GR) und ihre Bedeutung für die Umwelt-, Ernährungs- und Wirtschaftsgechichte während der alpinen Bronzezeit*. Inauguraldissertation, Zur Erlangung der Würde eines Doktors der Philosophie vorgelegt der Philosophisch naturwissenschaftlichen Fakultät der Universität Basel: Brugg.

Stampfli HR. 1976. *Osteo-archäologische Untersuchung des Tierknochenmaterials der spätneolithischen Ufersiedlung-Auvernier La Saunerie nach den Grabungen 1964 und 1965*: Solothurn.

Wettstein E. 1924. Die Tierreste aus dem Pfahlbau am Alpenquai in Zürich. *Vierteljahrsschrift der Naturforschenden Gesellschaft in Zürich* **69**: 78−127.

**Appendix S2**. References used for dating the assemblages listed in Table S1.

Bleicher N. 2011. Absolutdatierung. In *Zürich <Mozartstrasse> Neolithische und bronzezeitliche Ufersiedlungen 4, Die frühbronzezeitliche Besiedlung, Monographien der Kantonsarchäologie Zürich 42.* Schmidheiny M (ed.). Zürich und Egg; 49−67.

David-El Biali M. 1990. L’âge du Bronze en Valais et dans le Chablais vaudois: Un état de la recherche. *Jahrbuch der Schweizerischen Gesellschaft für Ur- und Frühgeschichte 73*, Huber & Co.: Frauenfeld; 19−50 (44−50 described by Chaix L).

Gross-Klee E. 1997. Einleitung. In *Ökonomie und Ökologie neolithischer und bronzezeitlicher Ufersiedlungen am Zürichsee, Ergebnisse der Ausgrabungen Mozartstrasse, Kanalisationssanierung Seefeld, AKAD/Pressehaus und Mythenschloss in Zürich*, Schibler J, Hüster Plogmann H, Jacomet S, Brombacher C, Gross-Klee E, Rast-Eicher A (eds.). Monographien der Kantonsarchäologie Zürich 20: Zürich und Egg; 13−39.

Hild A. 1935. Wallsiedlung im Borscht, Grabung 1935. *Jahrbuch des Historischen Vereins für das Fürstentum Liechtenstein*, Fünfunddreizigster Band, Im Selbstverlage des Dereins: 95−113.

Hild A. 1936. Wallsiedlung im Borscht, Grabung 1936. *Jahrbuch des Historischen Vereins für das Fürstentum Liechtenstein*, Fünfunddreizigster Band, Im Selbstverlage des Dereins: 31−49.

Hochuli S, Niffeler U, Rychner V. 1998. *SPM III Bronzezeit*, Die Schweiz vom Paläolithikum bis zum frühen Mittelalter Vom Neandertaler bis zu Karl dem Grossen, Verlag Schweizerische Gesellschaft für Ur- und Frühgeschichte: Basel.

Keller F. 1870. *Einige Bemerkungen über die Flugschrift des Herrn Aeppli, Lehrer von Obermeilen, betreffend die Entdeckung der Pfahlbauten daselbst*. Druck von J. Herzog: Zürich.

Kuhn E. 1935. Die Fauna des Pfahlbaues Obermeilen am Zürichsee. *Vierteljahrsschrift der Naturforschenden Gesellschaft in Zürich* **80**; 65−154.

Murbach-Wende I. 2016. *Cazis, Cresta: Die Keramik*, Archäologie Graubünden Sonderheft 5, Archäologischer Dienst Graubünden/Amt für Kultur, Somedia Buchverlag: Glarus/Chur.

Rageth J. 1986. Die wichtigsten Resultate der Ausgrabungen in der bronzezeitlichen Siedlung auf dem Padnal bei Savognin (Oberhalbstein GR). *Jahrbuch der Schweizerischen Gesellschaft für Ur- und Frühgeschichite 69*, Huber & Co: Frauenfeld; 63−103.

Stöckli WE, Niffeler U, Gross-Klee E. 1995. *SPM II Neolithikum*, Die Schweiz vom Paläolithikum bis zum frühen Mittelalter Vom Neandertaler bis zu Karl dem Grossen, Verlag Schweizerische Gesellschaft für Ur- und Frühgeschichte: Basel.

Wyss R. 2002. *Archaeologische Forshungen, Die bronzeueitliche Hügelsiedlung Cresta bei Cazis, Ergebnisse der Grabungen von 1943 bis 1970. Band 1 Teil I Die Siedlungen, Teil II Die Kleinfunde (ohne Keramik)*, Schweizerisches Landesmuseum: Zürich.

**Appendix S3**. References used for culture in Table S2.

Rychner V. 1998a. Geschichze und Kulturen der Bronzezeit in der Schweiz. In *SPM III Bronzezeit: Die Schweiz vom Paläolithikum bis zum frühen Mittelalter. Vom Neandertaler bis zu Karl dem Grossen*, Hochuli S, Niffeler U, Rychner V (eds.). Verlag Schweizerische Gesellschaft für Ur- und Frühgeschichte: Basel; 103−133.

Stöckli E. 1995. Geschichte des Neolithikuns in der Schweiz. In *SPM II Neolithikum: Die Schweiz vom Paläolithikum bis zum frühen Mittelalter. Vom Neandertaler bis zu Karl dem Grossen*, Stöckli S, Niffeler U, Gross-Klee E (eds.). Verlag Schweizerische Gesellschaft für Ur- und Frühgeschichte: Basel; 19−52.
