# Supplementary figures and images for: Size diversity in Swiss Bronze Age cattle
Source: Int J Osteoarchaeol. 2018 Apr 17;28(3):294–304. doi: 10.1002/oa.2654 (PMC6032853; doi:10.1002/oa.2654)

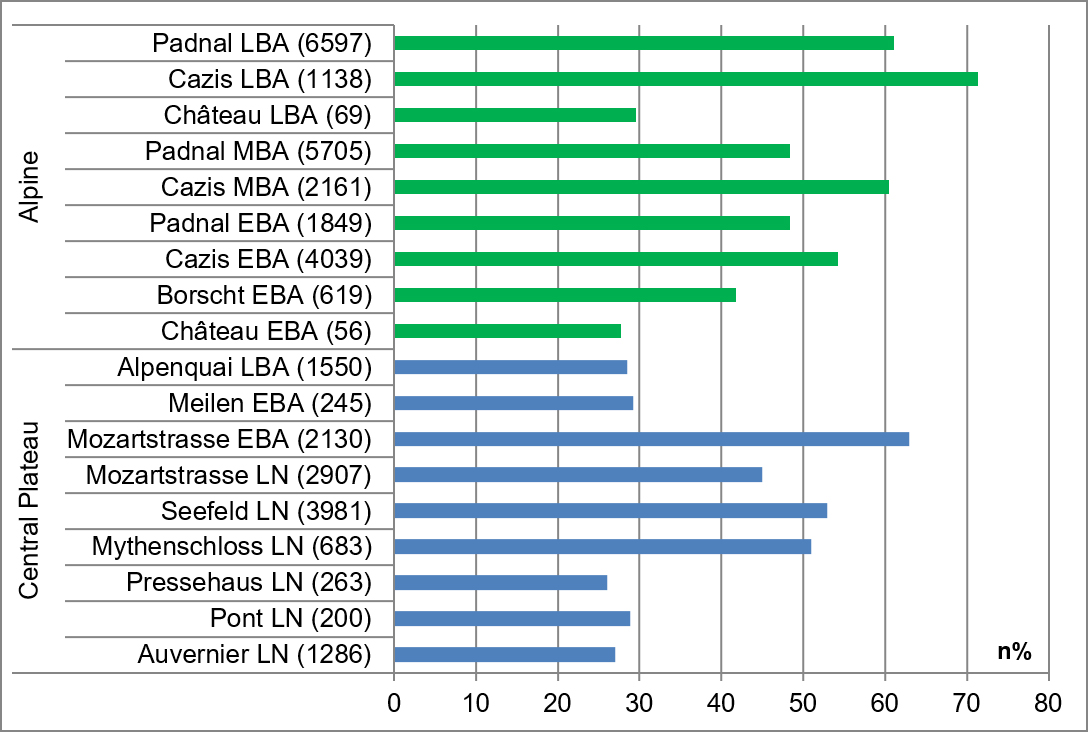

Supplement: Supplementary file 1 — Figure S1. Cattle proportion. Figure S2. Cattle mortality profile. Table S1. Dating of the assemblages. Table S2. Detailed list of the studied sites. Table S3. Statistical summary for Figure 2 and 3. Table S4. Statistical summary for Figure 4. Table S5. Statistical summary for Figure 7. Appendix S1. References for Table 1. Appendix S2. References for Table S1. Appendix S3. References for Table S2. [file OA-28-294-s001.zip › Supplementary Figure S1.jpg]

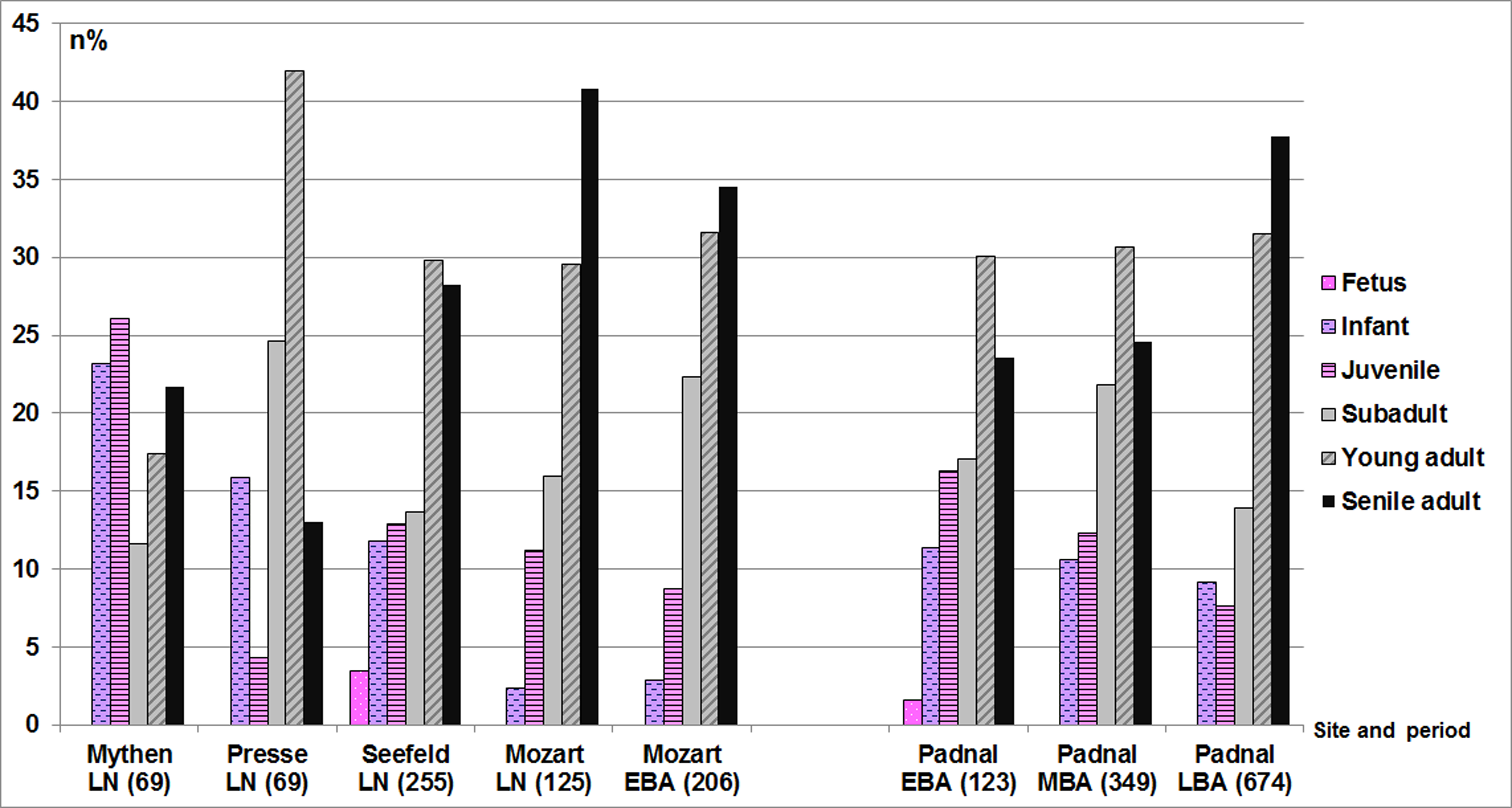

Supplement: Supplementary file 1 — Figure S1. Cattle proportion. Figure S2. Cattle mortality profile. Table S1. Dating of the assemblages. Table S2. Detailed list of the studied sites. Table S3. Statistical summary for Figure 2 and 3. Table S4. Statistical summary for Figure 4. Table S5. Statistical summary for Figure 7. Appendix S1. References for Table 1. Appendix S2. References for Table S1. Appendix S3. References for Table S2. [file OA-28-294-s001.zip › Supplementary Figure S2.jpg]
